# Efficient Hydrophobic Modification of Old Newspaper and Its Application in Paper Fiber Reinforced Composites

**DOI:** 10.3390/polym11050842

**Published:** 2019-05-10

**Authors:** Weiwei Zhang, Jin Gu, Dengyun Tu, Litao Guan, Chuanshuang Hu

**Affiliations:** College of Materials and Energy, South China Agricultural University, Guangzhou 510642, China; zhangww@scau.edu.cn (W.Z.); gujin@scau.edu.cn (J.G.); tudengyun@scau.edu.cn (D.T.); ltguan@scau.edu.cn (L.G.)

**Keywords:** ONP fibers, silanization, composites, mechanical properties

## Abstract

Paper fibers have gained broad attention in natural fiber reinforced composites in recent years. The specific problem in preparing paper fiber reinforced composites is that paper fibers easily become flocculent after pulverization, which increases difficulties during melt-compounding with polymer matrix and results in non-uniform dispersion of paper fibers in the matrix. In this study, old newspaper (ONP) was treated with a low dosage of gaseous methyltrichlorosilane (MTCS) to solve the flocculation. The modified ONP fibers were characterized by Scanning Electron Microscope (SEM), Fourier Transform Infrared Spectroscopy (FTIR), and Thermogravimetric Analysis (TG). Then the modified ONP fibers and high-density polyethylene (HDPE) were extruded and pelletized to prepare ONP/HDPE composites via injection molding. Maleic anhydride-grafted polyethylene (MAPE) was added to enhance the interfacial bonding performance with the ultimate purpose of improving the mechanical strength of the composites. The mechanical properties such as tensile, flexural, and impact strength and the water absorption properties of the composite were tested. The results showed that the formation of hydrogen bonding between ONP fibers was effectively prevented after MTCS treatment due to the reduction of exposed –OH groups at the fiber surface. Excessive dosage of MTCS led to severe fiber degradation and dramatically reduced the aspect ratio of ONP fibers. Composites prepared with ONP fibers modified with 4% (*v*/*w*) MTCS showed the best mechanical properties due to reduced polarity between the fibers and the matrix, and the relatively long aspect ratio of treated ONP fibers. The composite with or without MAPE showed satisfactory water resistance properties. MTCS was proven to be a cheap and efficient way to pretreat old newspaper for preparing paper fiber reinforced composites.

## 1. Introduction

Natural fibers, as an alternative to conventional reinforcement in polymer composites, have received significant interest during the past decades [1,2]. Compared with synthetic fibers, natural fibers possess many advantages such as low cost, low density, CO_2_ neutral, sustainability, biodegradability, high specific strength/modulus, and being non-abrasive to processing machinery. In natural fiber reinforced composites, the compatibility between hydrophilic natural fibers and hydrophobic thermoplastics is the main problem to be solved since the interfacial properties play an important role in the physical and mechanical performances of the composites. Results showed that interfacial adhesion could be improved through physical treatments [3,4], chemical modification [5,6], or a combination of both [7,8] to natural fibers. Recently, researchers have focused on preparing composites with paper fibers [9,10,11,12,13,14,15,16,17]. Usually paper fibers have a length in the range of 500–5000 μm and a diameter of 10–50 μm. Their absolute length is shorter than that of hemp fiber but their aspect ratio is larger than that of wood powder fiber. Such properties make them suitable for fabricating reinforced composites with applications in construction, automobiles, and outdoor furniture. 

In the injection molding process, waste paper needs mechanical milling to produce individual fibers, followed by melt-compounding with thermoplastic polymers to disperse paper fibers uniformly within the composites. However, paper fibers are always flocculent after direct pulverization. Such flocs makes paper fibers difficult to melt-compound with the polymer matrix, which results in non-uniform dispersion of paper fibers in the matrix. Valente et al. [18] reported that paper turned prevalently wad-like after micronization and a turbo-mixing had been developed to introduce higher fiber percentages and better dispersion in the high-density polyethylene (HDPE) matrix. The agglomeration of paper fibers is mainly attributed to their relatively high aspect ratio and hydrogen bonding with each other. Therefore, methods that could reduce the formation of hydrogen bonds between fibers would facilitate their melt-compounding with polymer matrix. Serrano and Espinach et al. [19,20] used a water–diglyme (1:3) mixture to disperse old newspaper fibers before an oven drying process to limit the hydrogen bonding between the cellulosic fibers. Yang and Wang et al. [21] investigated several methods for dispersing pulp fibers in the HDPE matrix, and found that combining twice twin-screw melt-blending with pre-pressing and oven drying would obtain composites with the most uniform fiber dispersion and the best mechanical properties. The hydrophobic modification at fiber surface by acetylation [22,23], silane treatment [24,25], maleic anhydride treatment [6], etc., could also improve compatibility between the paper fibers and the polymer matrix through the reduction of fiber polarity. However, the concentration of paper fibers in the reaction system is usually kept as low as 2% or less to ensure that the chemical modification is uniform and sufficient, which meant the amount of organic solvents used was at least 50 times more than the paper fibers. Even for potassium methyl siliconate-treated pulp fibers, which were treated in water instead of organic solvents [26,27], 300 g fibers required 990 g potassium methyl siliconate for the modification process. It seemed that the solvent reaction system was not an efficient method for mass paper fiber modification.

Methyltrichlorosilane (MTCS) is an active silane coupling agent and has been successfully used to prepare superhydrophobic surfaces. Gao and McCarthy [28] prepared a perfectly hydrophobic surface with advancing and receding angles of 180° using an MTCS/toluene system. Water traces in this liquid reaction system were used to adjust the three dimensional nanostructured geometry and the hydrophobicity of the modified surface [29,30]. The silicone nanofilaments could also be formed in closed gaseous environments with controlled water vapor [31]. Superhydrophobic textiles and cellulosic products were fabricated by a one-step gas phase coating procedure using gaseous MTCS [32,33]. If the hydrophobic modification of paper fibers was carried out in a liquid solvent, 300 g fibers required 15 L toluene (with a fiber concentration of 2%) and 224 g MTCS (with a MTCS concentration of 0.1 mol/L). However, it only takes 15 g MTCS (nearly 15 times less) if the reaction happens in the gas phase and no organic solvents are required. Thus the gas phase reaction has the potential to be suitable for paper fiber hydrophobization. 

In this study, old newspaper (ONP) was recycled as a typical waste paper to prepare natural fiber composites. High-density polyethylene (HDPE) was chosen as the matrix in consideration of the relatively low processing temperature so that the degradation on ONP fibers from high temperature and high pressure in a twin-screw extruder could be minimized. The flocculation problem of paper fibers was solved by hydrophobic modification using MTCS. The effect of MTCS treatment on the fiber properties and mechanical properties of ONP–HDPE composites was investigated. FTIR results showed that some exposed –OH groups still existed at the fiber surface after MTCS modification, indicating that the interface compatibility between hydrophobic ONP fibers and HDPE matrix was still flawed. Therefore, maleic anhydride-grafted polyethylene (MAPE), a common coupling agent used in natural fiber–plastic composites [34,35,36], was further added into the composite to improve its physical and mechanical properties. 

## 2. Materials and Methods

### 2.1. Materials

Old newspaper (ONP) with an apparent density of 0.89 g/cm^3^ was provided by Nanfang Metropolis Daily, Guangzhou, China. The main components contained 46.9% cellulose, 24.0% hemicellulose, 16.5% lignin, and 10.9% ash. Its moisture content was measured to be 6.7% at room temperature and 60% relative humidity. Methyltrichlorosilane (MTCS, 98%) was supplied by J&K Chemical, Beijing, China. High-density polyethylene (HDPE, 5000S) pellets with a density of 0.954 g/cm^3^ and a melt flow index of 0.92 g/10 min (190 °C, 2.16 kg according to ASTM D1238) were purchased from Yangzi Petrochemical in Guangzhou, China. The tensile and flexural strength of pure HDPE was measured to be 19.6 and 12.2 MPa, respectively. Maleic anhydride-grafted polyethylene (MAPE) with an MA grafting ratio of 0.9 wt.% was supplied by Bochen Polymer New Materials, Foshan, China. The free radical grafting reaction happened between MA and HDPE at around 170 °C.

### 2.2. ONP Pretreatment

In a typical pretreatment, one batch of ONP (100 g) was cut into 4 mm × 25 mm strips using a paper shredder and dried at 105 °C for 2 h. The dried ONP strips were sealed in a plastic bag containing a dish with a certain volume of MTCS. The MTCS dosages of 2, 4, 6, and 8 mL were marked as 2%, 4%, 6%, and 8% (*v*/*w*), respectively. The bag was sealed and placed in an oven at 60 °C for 3 h. The liquid silane completely volatilized during this period. The bag was then opened and 20 mL of water was added into the dish. The bag was sealed and placed in an oven at 60 °C for another 3 h to hydrolyze the residual MTCS. The treated ONP fibers were washed with deionized water until they were pH neutral. Then they were oven-dried at 120 °C for 2 h. Three batches of the powdery fibers (300 g) were mixed with HDPE pellets (300 g, including MAPE pellets) in a high-speed mixer (SHR-10A, Grand Instrument Co. Ltd., Zhangjiagang, China) at 100 °C for 10 min. The well-mixed mixture was stored in sealed bags for further extrusion–pelletization processes. 

### 2.3. Preparation of Composites 

ONP/HDPE composites were prepared through extrusion–pelletization and injection molding. The melt-compounding and pelleting was carried out in a twin-screw extruder (SHJ-20B, Jieente, Nanjing, China) with an *L*/*D* ratio of 40. The temperatures of five zones in the extruder were set to be 130, 135, 155, 150, and 145 °C, respectively and the rotation speed was 16 rpm. The extruded cylindrical sample with a diameter of 3.5 mm, was cut by a granular QL-20 (SHJ-20B, Jieente, Nanjing, China) to produce pellets with a length of 5 mm. The pelletized composites were dried at 105 °C for 4 h before feeding into an injection molding machine (Y-35V, Yingbao Instrument Co. Ltd., Dongguan, China) for preparing the specimens for the testing of mechanical properties. The injection temperature was set to be 170 °C, injection pressure 95 bar, injection time 2 s and cooling time 4 s.

### 2.4. Characterization

#### 2.4.1. Mechanical Properties

All test specimens were kept at 25 °C and 65% relative humidity for seven days before testing. Tensile and flexural properties were measured using an electromechanical universal testing machine (Model CMT5504, Shenzhen Rethink Cooperation, Shenzhen, China) according to GB/T 1040-2006 and GB/T 9341-2008, respectively. A dumbbell specimen was used for measuring the tensile strength and the loading speed was 5 mm/min. A flexural specimen, with the dimensions of 80 mm × 10 mm × 4 mm, was used for measuring the flexural strength and flexural modulus. The loading speed was 2 mm/min. The notched impact strength was measured by a load impact testing machine (XJU-5.5, Chengde Xinma Testing Instrument Co. Ltd., Chengde, China) according to GB/T 1843-2008. Notch depth of the impact specimen was 0.25 mm. Five replicates were tested to obtain the average values of the above mechanical properties and their standard deviations. 

#### 2.4.2. Water Absorption Tests

The water resistance test was conducted according to GB/T 1934.1-2009. All the specimens were oven dried at 50 ± 3 °C for 24 h prior to water soaking and then immersed in water for 24 h at room temperature. Each specimen was weighed and measured for thickness at three marked locations. Five replicates were tested for each specimen.

#### 2.4.3. Scanning Electron Microscope (SEM) 

The morphologies of the modified ONP fibers and the fracture surfaces of the composites were observed with an SEM (Hitachi S-4800, Hillsboro, OR, USA) at an accelerating voltage of 20 kV. All samples were sputter-coated with gold before SEM observation. 

#### 2.4.4. Wettability Test

The wettability of original and MTCS-modified ONP fibers was characterized by water contact angle (CA) using Contact Angle System OCA20 (DataPhysics, Filderstadt, Germany). Flocculent and powdery ONP fibers were adhered to a glass sheet using double-sided tape to form a relative flat film. Then graphs of 5 μL water droplets on the film were recorded and their CAs were calculated by Young–Laplace fitting. 

#### 2.4.5. Fourier Transform Infrared Spectroscopy (FTIR) 

Fourier transform infrared analysis of untreated and treated ONP fibers was carried out in a Fourier transform infrared spectrometer (Tensor-27, Bruker, Ettlingen, Germany). Each sample was scanned 64 times in transmittance mode, at 4 cm^−1^ resolution in the wave range from 400 to 4000 cm^−1^.

#### 2.4.6. Thermogravimetric Analysis

Thermogravimetric analysis was used to characterize the thermal stability of the modified ONP fibers with a TG 209 Instrument (Bruker Netzsch, Selb, Germany) at a heat rate of 10 °C/min from room temperature to 700 °C under nitrogen gas. 

## 3. Results and Discussion 

### 3.1. Characterization of Untreated and MTCS-Treated ONP Fibers

**Figure 1:**
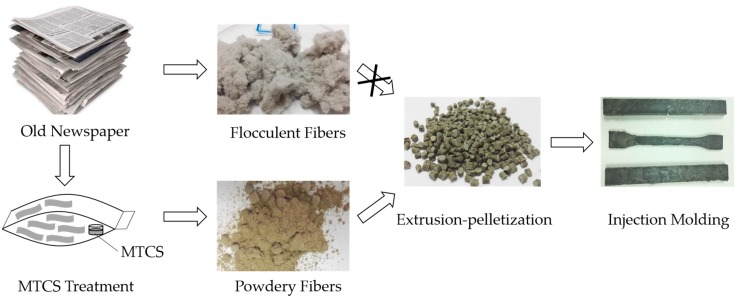
Schematic diagram of old newspaper (ONP)/high-density polyethylene (HDPE) composite preparation.

Direct mechanical pulverization of ONP without any treatment produced flocculent fibers as seen in Figure 1. These ONP fibers could not be fed into the twin-screw extruder for melt-compounding and pelleting with HDPE pellets. The modified ONP fibers pre-treated with MTCS were powdery and did not need further mechanical pulverization, which indicated that hydrogen bonding between fibers was effectively prevented. Thus ONP powder could be smoothly melt-compounded with HDPE via a twin-screw extruder, followed by injection molding.

**Figure 2:**
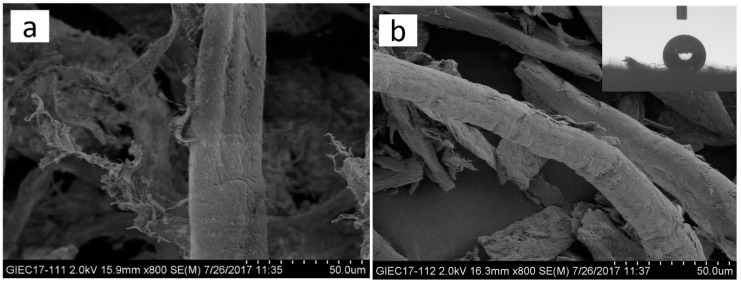

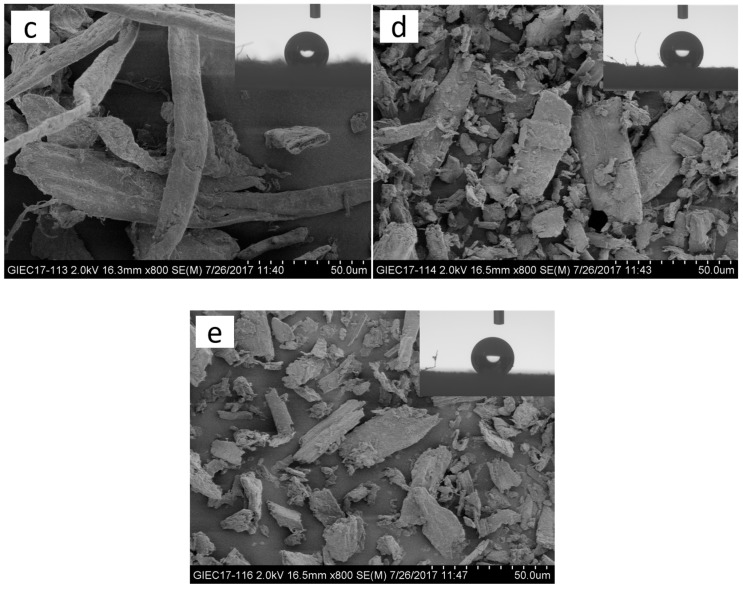
SEM images of ONP fibers treated with different methyltrichlorosilane (MTCS) dosages and their corresponding water contact angle photos: (**a**) 0 (*v*/*w*); (**b**) 2% (*v*/*w*); (**c**) 4% (*v*/*w*); (**d**) 6% (*v*/*w*); and (**e**) 8% (*v*/*w*).

The surface morphology of untreated and MTCS-treated ONP fibers was characterized using SEM. It can be observed from the SEM images shown in Figure 2 that there were no obvious changes at the fiber surface treated with 2% and 4% (*v*/*w*) MTCS compared with the untreated ONP fibers shown in Figure 2a. A monolayer of polymethylsilsesquioxane (PMS) was formed at the modified fiber surface because MTCS reacted with the cellulose –OH groups in the sealed dry environment [37]. A few fibrous fragments were observed when 2% and 4% (*v*/*w*) MTCS was used. However, no intact fibers were found when the MTCS dosage increased to 6% and 8% (*v*/*w*), and the aspect ratio of fiber debris decreased significantly to two or even smaller. The dimension of the powdery fiber decreased with the increase of the MTCS dosage. The degradation of ONP fibers was attributed to the presence of the by-product hydrogen halide from the MTCS hydrolysis. More severe degradation of the ONP fibers was observed when hydrogen halide concentration increased with the increased usage of MTCS during silanization modification. 

The wettability of MTCS-modified ONP fibers was investigated through measuring their water contact angles. Before modification, a water droplet penetrated into ONP fibrous film within 5 s. Due to the introduction of Si–CH_3_ groups at the ONP fiber surface, the water contact angle of the ONP fibers after treatment with 2% and 4% (*v*/*w*) dosage of MTCS increased to 129.1° ± 6.0° and 129.9° ± 3.8°, respectively. With the increase of the dosage of MTCS, the hydrophobicity of modified ONP fibers was further enhanced and the contact angles went up to 138.6° ± 3.2° for 6% (*v*/*w*) and 139.8° ± 0.3° for 8% (*v*/*w*). MTCS modification turned ONP fibers from hydrophilic to hydrophobic, which demonstrated that their interface compatibility would be improved with HDPE matrix. 

**Figure 3:**
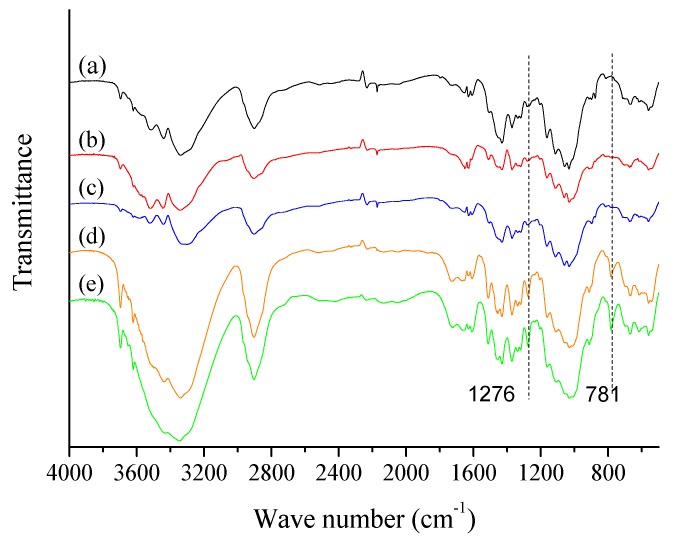
FTIR spectra of ONP fibers treated with different MTCS dosages: (a) 0 (*v*/*w*); (b) 2% (*v*/*w*); (c) 4% (*v*/*w*); (d) 6% (*v*/*w*); and (e) 8% (*v*/*w*).

FTIR spectra shown in Figure 3 were used to characterize the changes of the chemical groups that occurred at the treated ONP fibers. There existed the overlapping effects of C–O in cellulose and Si–O–Si, Si–O–C, which were derived from the reaction between the hydroxyl groups of fibers and MTCS at 1000–1130 cm^−1^ [38]. Characteristic absorption peaks at the 781 and 1276 cm^−1^ were assigned to the Si–C stretching vibration bond, and the deformation vibrations of –CH_3_. However, these two peaks were only found in the 6% and 8% (*v*/*w*) MTCS-treated samples, which indicated the presence of PMS. Meanwhile the two characteristic absorption bands at 781 and 1276 cm^−1^ were not obvious in samples treated with 4% (*v*/*w*) MTCS, which supports that the modified fiber surface was covered with only a small amount of PMS. The broad peaks at 3200–3680 cm^−1^ and 2900 cm^−1^ were assigned to the stretching vibration of –OH and CH_2_ groups from cellulose and they decreased with the increase of MTCS dosage to 4% (*v*/*w*). However, these bands became pronounced again in samples treated with the 6% (*v*/*w*) and 8% (*v*/*w*) MTCS. It was thought that more fiber fragmentation under these two conditions possibly exposed new hydrophilic hydroxyl groups within the ONP fibers. 

**Figure 4:**
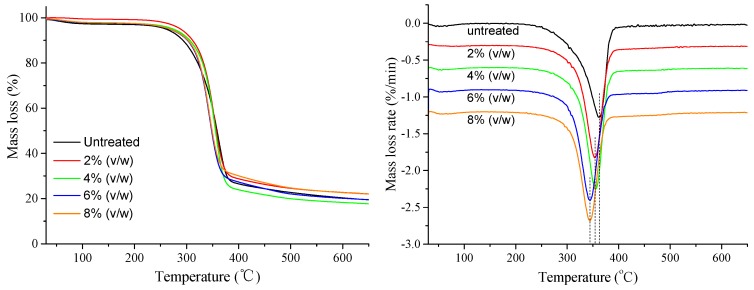
TG spectra of untreated and modified fibers with 2%, 4%, 6%, and 8% (*v*/*w*) MTCS dosages.

The thermal stability of untreated and MTCS-modified ONP fibers was studied by TGA as shown in Figure 4. Usually, natural fibers exhibited three stages of mass loss under nitrogen atmosphere with increasing temperature. The initial stage took place before 120 °C due to the evaporation of adsorbed moisture. The second stage corresponded to swift decomposition between 275 and 370 °C, which was thought to be hemicellulose and partly cellulose degradation. An unsymmetrical peak at 362 °C in untreated ONP fibers was observed while the peak symmetry of MTCS-modified fibers was improved, indicating that partial hemicellulose was removed after the MTCS treatment and washing process. However, the degradation temperature at maximum rate shifted to 355 and 344 °C, corresponding to 2%, 4% (*v*/*w*) and 6%, 8% (*v*/*w*) MTCS treatment, respectively. The lower cellulose-breakdown temperature showed a degradation of thermal stability of the modified ONP fibers. The thermal stability degradation probably resulted from the HCl degradation of ONP fibers, which was consistent with information shown in the SEM images. 

**Figure 5:**
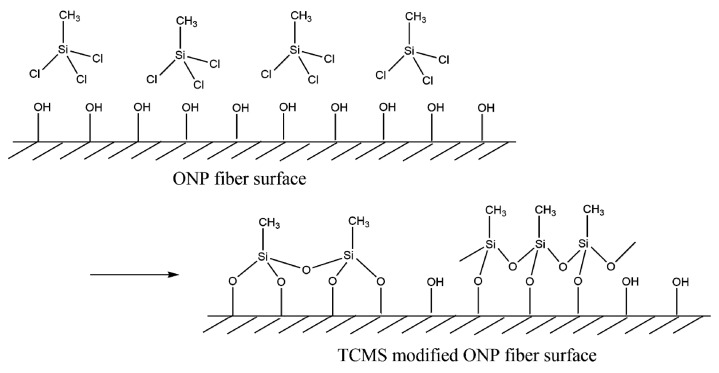
Formation of polymethylsilsesquioxane (PMS) on ONP fiber surface.

Based on the above characterization of MTCS-modified ONP fiber, the possible reaction mechanism is proposed in Figure 5. Gaseous MTCS could readily be impregnated into ONP strips through the interspaces. Since no free water existed on the ONP fibers, these reactive –Cl groups of MTCS assembled on the fiber surfaces and reacted with –OH group of cellulose to form Si–O–cellulose bonds. Such bonds per silane molecule reached at maximum two instead of three due to the steric hindrance [39]. However, no condensation took place among free- or anchored-MTCS molecules due to the absence of water molecules. Hydrolysis reaction of residue Si–Cl groups happened in the next step where water was introduced. Therefore, a monolayer of PMS was finally formed at the ONP fiber surface, producing hydrophobic properties. Note that the PMS layer was not complete as some –OH groups were preserved at the fiber surface after MTCS modification. 

### 3.2. Effect of MTCS Dosage on Mechanical Properties and Water Resistance of ONP/HDPE Composites

Powdery ONP fibers were obtained after MTCS treatment due to the reduced polarity and decreased hydrogen bonding among ONP fibers. Macro-morphology transition of ONP fibers was essential because flocculent fibers could not be fed into the twin-screw extruder for melt-compounding and pelleting with HDPE pellets. 

**Figure 6:**
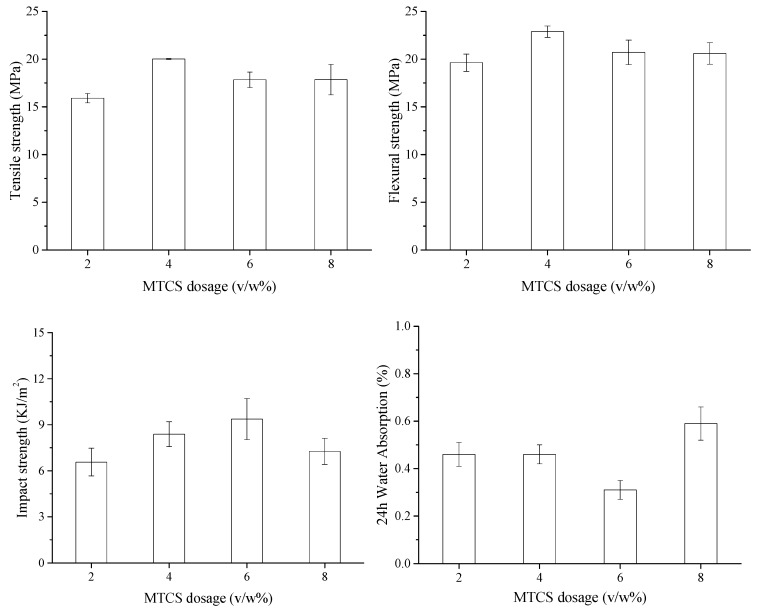
Impacts of MTCS dosages on mechanical properties and water resistance of ONP/HDPE composites.

The relationship between the MTCS dosages and the mechanical properties of ONP/HDPE composites was investigated and the results are shown in Figure 6. The impacts of MTCS dosage on tensile and flexural strength were similar. The increase of tensile and flexural strengths with the increase of MTCS dosage from 2% to 4% (*v*/*w*) could be attributed to the improved compatibility between the modified fibers and HDPE. The fiber orientation and the degree of dispersion might be also enhanced due to weakened forces between the modifier fibers [40]. However, tensile and flexural strengths fluctuated when the MTCS dosage was larger than 4% (*v*/*w*), which might be caused by the compromise between decreased aspect ratio and improved compatibility. Although the notched impact strength also increased at first with the increase of MTCS dosage and then decreased, the highest notched impact strength was obtained when the MTCS dosage was 6% (*v*/*w*). The SEM images showed that the modified ONP fibers were dramatically damaged with a relatively high dosage of MTCS. Fibers treated with 8% (*v*/*w*) MTCS even appeared irregularly shaped. The reduced aspect ratio led to more fibers pulled out of the matrix rather than fracture when the ONP/HDPE composite failed to reduce its mechanical strength [9]. It seemed that impact strength was less sensitive to the aspect ratio of paper fiber reinforcement than tensile and flexural strength. 

Water absorption of ONP/HDPE composites was low and decreased with the increased dosage of MTCS up to 6% (*v*/*w*). The prepared composites showed worse water resistance when large amounts of MTCS were used to modify the ONP fibers. As mentioned above, ONP fibers treated with 8% (*v*/*w*) MTCS were intensively fragmented, which exposed more hydrophilic hydroxyl groups within the ONP fibers and would result in more interfacial bonding and more moisture uptake. 

### 3.3. Effect of MAPE Content on Mechanical Properties and Water Resistance of ONP/HDPE Composites

**Figure 7:**
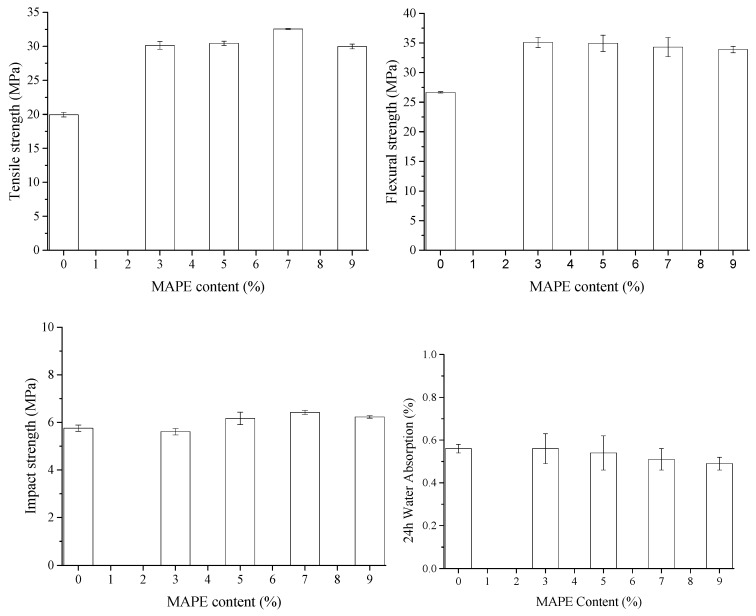
Impacts of maleic anhydride-grafted polyethylene (MAPE) content on mechanical properties and water resistance of ONP–HDPE composites.

The hydrophobic modification at the ONP fibers improved interfacial compatibility with HDPE matrix. However, the promoted mechanical properties of the composites were limited. There were still large amounts of free hydroxyl groups at the MTCS-treated fibers according to the information obtained from the FTIR results. Therefore, the coupling agent MAPE was added during the extrusion–pelletization process to improve the interface bonding and to enhance the mechanical properties. The presence of MAPE in the composites created a significant increase in tensile strength, flexural strength, and notched impact strength as shown in Figure 7. The tensile strength of the composite quickly increased with 3 wt.% of MAPE and after that slowly reached the highest value of 32.57 ± 0.07 MPa, which was up to a 63.2% enhancement compared to the one without MAPE. The flexural strength quickly increased when the MAPE content was 3 wt.% and then decreased linearly from 35.08 ± 0.88 to 33.89 ± 0.54 MPa when the MAPE content increased from 3 to 9 wt.%. The notched impact strength of the ONP/HDPE composite was less affected by the addition of MAPE. The strengthening effect of MAPE on the ONP/HDPE composites can be attributed to the ester linkages of the unreacted hydroxyl groups on the fibers and the acid anhydride groups of MAPE while the nonpolar long chain from MAPE was entangled with the molecular chain of the matrix. The improved interface adhesion between the ONP fibers and HDPE matrix also reduced the water absorption of composites. Since the ONP fibers turned hydrophobic after MTCS modification, the 24 h water absorption of the ONP/HDPE was as low as 0.56% ± 0.02%. Thus, the increase of MAPE content only slightly reduced the water absorption behavior of the ONP/HDPE composites.

### 3.4. Interfacial Characterization of ONP/HDPE Composites

**Figure 8:**
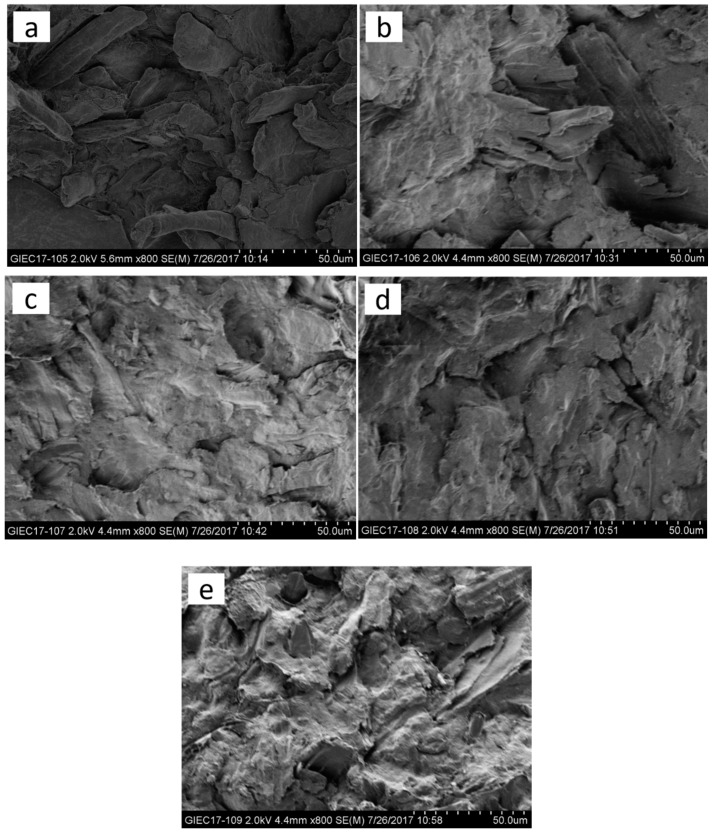
SEM images of interfacial adhesion with different MAPE contents: (**a**) 0%; (**b**) 3%; (**c**) 5%; (**d**) 7%; and (**e**) 9%.

The interfacial adhesion of ONP and HDPE could be inferred from the fracture surface microstructure of ONP/HDPE composite. SEM images of fracture surfaces of the composites reinforced with 50% fibers which were treated with 4% (*v*/*w*) MTCS are shown in Figure 8. A composite prepared without MAPE showed identifiable ONP fibers as well as gaps around them, which indicated that the interfacial adhesion between modified fibers and the matrix was weak. The reason was thought to be that the polarity difference between the modified ONP fibers and HDPE had decreased but not disappeared, since cellulose –OH groups still existed at the modified fiber surface with 4% (*v*/*w*) MTCS according to the FTIR results. The interface of ONP and HDPE became more and more ambiguous and no debonding of fibers was observed with increased MAPE loading shown in Figure 8b–e. The interfacial adhesion was enhanced since carboxyl groups of MAPE connected with hydroxyl groups of ONP fibers through ester and/or hydrogen bonding.

## 4. Conclusions

In this study, hydrophobic modification using gaseous MTCS was proved to be an efficient method for ONP to solve the flocculation problem after pulverization. The formation of hydrogen bonding between ONP fibers was effectively prevented after MTCS treatment due to the reduction of exposed –OH groups at the fiber surface. However, excessive dosage of MTCS (larger than 6% (*v*/*w*)) led to severe fiber degradation and dramatically reduced the aspect ratio of ONP fibers. ONP/HDPE composites prepared with ONP fibers modified with 4% (*v*/*w*) MTCS showed the best mechanical properties due to reduced polarity between fibers and matrix, and the relatively long aspect ratio of treated ONP fibers. The composite with or without MAPE showed satisfactory water resistance properties. The interfacial bonding performance between ONP fibers and HDPE matrix as well as the physical and mechanical properties of the composites was further enhanced with the assistance of the coupling agent MAPE.
